# Graves' Disease Associated with Cerebrovascular Disease and Antiphospholipid Antibody Syndrome

**DOI:** 10.1155/2010/624152

**Published:** 2010-09-02

**Authors:** Ines Khochtali, Nadia Hamza, Elyes Gassab, Asma Baba, Maha Kacem, Mahbouba Frih, Sylvia Mahjoub

**Affiliations:** ^1^Endocrinology Unit, Department of Internal Medicine,, Monastir Hospital, Monastir 5000, Tunisia; ^2^Department of Ear, Nose and Throat, Monastir Hospital, Monastir 5000, Tunisia; ^3^Department of Neurology, Monastir Hospital, Monastir 5000, Tunisia

## Abstract

Thyroid disorders are commonly associated with coagulopathy. Patients with hyperthyroidism have increased risk for developing thromboembolic accidents, which are favoured by a simultaneous presence of antiphospholipid antibodies syndrome. in this paper, we describe the case of a patient with Graves' disease, who developed strokes with antiphospholipid antibodies syndrome.

## 1. Introduction

 Hyperthyroidism is associated with an increased risk of thromboembolic complications. The association between Graves' disease and ischemic stroke is very rare. It might be due to different aetiologies. The authors wish that the discussion here in may have influence on treatment choice. 

## 2. Observation

We report a case of 39-year-old man without familial history of thyroid dysfunction, autoimmune disease, or thrombosis. 

He presented with an acute right hemiplegia for which he was hospitalized in neurology department. Computed tomography (CT) scan had confirmed the presence of lacunar infarction in the internal capsule of the left cerebral hemisphere. 

He was referred to our endocrinology unit because of signs of thyrotoxicosis including palpitation, polydipsia, and significant weight loss (6 kilograms).

Physical examination revealed a body mass index of 25 kg/m², a blood pressure within normal range of 130/80 mm Hg, heart rate of 96 beats/min, bilateral exophthalmos, homogeneous goitre, and right hemiparesis. 

The electrocardiogram showed regular sinus rhythm without atrial fibrillation.

Thyroid function studies revealed undetectable serum thyroid-stimulating hormone (TSH) (below 0.05 mUI/L) and positive antithyroid-stimulating hormone receptor antibodies confirming the diagnosis of Graves' disease (Table 1).

Thyroid scan with technetium 99 m (Tc-99 m) showed an enlarged thyroid gland with diffuse increased uptake.

Fasting blood glucose was 14.3 mmol/L and remained high in the subsequent assessment confirming the presence of diabetes, according to World Health Organization. The antibodies to glutamic acid décarboxylase (GAD) were negative. 

For the assessment of his cerebrovascular accident, other investigations were performed showing positive antiphospholipid (APL) antibodies with IgG anti-*β*2-Glycoprotien-I positive (*β*2GP-I) and IgM anticardiolipin antibody positive which remains positive 3 months later (Table 1).

Thrombophilic factors including protein C activity, antithrombin III, protein S, and prothrombin time were within normal range. Antinuclear antibodies were negative.

The diagnosis of Graves' disease associated with a primary antiphospholipid syndrome (APS) was confirmed. The patient was treated with Aspirin (250 mg/day) and benzyl thiouracil (25 mg) at the dose of 12 tablets/day, with progressive regression. Improvement was shown in clinical symptoms and laboratory studies; Glycaemia levels and glycated haemoglobin returned to normal without any antidiabetic treatment. 

## 3. Discussion

The association between cerebrovascular disease and Graves' disease is very rare. Sometimes, the cerebral arterial thrombosis can be explained by rhythm disorders like atrial fibrillation that is frequent in Graves' disease. This disorder is present in 9% to 22% of the cases of hyperthyroidism compared with 0.4% in the general population. It can reveal the hyperthyroidism; and 15 percent of strokes occur in people with atrial fibrillation [1].

It is also being increased in cases of preexistent heart disorder or in preferential hypersecretion of T3 [2]. Our patient had normal sinus rhythm in electrocardiogram. 

The cerebral symptoms could be explained by autoimmune encephalopathy. But the patients are usually euthyroid or hypothyroid with high antibody titers. Patients show a mild to moderate elevation of cerebrospinal fluid protein levels; rarely findings are suggestive of demyelination, such as oligoclonal bands and myelin basic protein.

The clinical picture is presented with variable symptoms from behavioral and cognitive changes, myoclonus, pyramidal tract dysfunction, and cerebellar signs to psychosis and coma, with relapsing and progressive course. The diagnosis is often overlooked at presentation but it is crucial [3]. In our case, we found right hemiplegia in the exam and left lacuna infarct in computed tomography without clinical and laboratory signs of autoimmune cerebral vasculitis.

During the hyperthyroidism, the influence of thyroid hormone on the coagulation-fibrinolytic system is mediated by the interaction between the hormone and its receptors; various abnormalities have been described, ranging from subclinical abnormalities to major hemorrhages or fatal thromboembolic events. Various changes in the coagulation-fibrinolytic system have been described in patients with an excess of thyroid hormones. An increased risk of thrombosis is found in hyperthyroidism [4, 5].

The Carotid artery dissection is a cause of ischemic stroke in young people; the possibility that a disorder of immunity might have a role in the mechanism of inflammatory alterations has been recently suggested. The hypothesis of an association between carotid artery dissection and thyroid disease has been suggested in few case reports [6, 7]. 

Our patient did not have headache or neck pain, and the neuroimaging did not show dissection of carotid vessels. 

The stroke in a young patient with Graves' hyperthyroidism can be caused by MoyaMoya disease. It caused multiple intracranial stenoses although this patient had a focal neurologic deficit and a single lacunar infarction on CT scan [8, 9].

Thyrotoxicosis, probably through a factor VIII-mediated hypercoagulability, may be a predisposing factor for cerebral venous thrombosis [10].

The complex haemostatic balance can be influenced by autoimmune mechanisms such as APS, but this rarely occur. 

*Graves' disease can be associated with APS. *The APS occurs at the presence of at least one of the clinical criteria and one of the laboratory criteria that are present in Table 2 [11].

This syndrome may arise in autoimmune disease mainly in the systemic lupus erythematosus or may occur de novo in which case it is known as primary APS.

*Our patient had primary APS. *These antibodies may lead to a number of clinical syndromes including venous and arterial thrombosis [12, 13]. 

In the literature, there are few cases published with association between APS and Graves' disease [4, 14–16]. 

The first case is a 48-year-old woman having recurrent venous thrombosis due to primary APS associated with hyperthyroidism. We noted the presence of Human Leukocyte Antigen (HLA) DR7 and a high rate of anti-TSH receptors. As for her hyperthyroidism, she was treated with carbimazole and propranolol; As for the APS, she was given heparin rather than coumadin with recanalization of her thromboses [4]. 

The second is of a 33-year-old woman known as having Graves' disease; she subsequently developed a cerebrovascular disease which had been explained by the increase of APL antibodies [14].

On immunogenetic analyses of families presenting with auto-immune diseases and APS, we demonstrated that the presence of the class HLA DR4 and DR7 predisposes the patients having Graves' disease to develop APS [17].

Other possibilities are advanced to demonstrate the relation between the APS and Graves' disease. The first possibility is suggesting the existence of a crossing reaction between *β*2-glycoprotein-I and the epitope of TSH receptors. The second possibility is that anti-TSH receptors antibodies can generate antibodies to epitopes of antiphospholipids [17, 18]. 

## 4. Conclusion

The association between APS and Graves' disease is very rare. It may have a dangerous consequence like cerebrovascular disease. We suggest that patients with Graves' disease should be examined for the presence of APS, especially when there is thrombosis accident.

## Figures and Tables

**Table 1:** Biological characteristic of the patient.

	Patient	Normal range
FT4	32.09	9–19 ng/l
TSH	<0.005	0.5–4.5 m UI/l
Anti-TSH receptor antibodies	12	<2 UI/ml
Antithyroglobulin antibodies	194	<50 UI/l
Antithyroperoxydase antibodies	1534	<50 UI/l
Anticardiolipin antibodies (IgM)	41–45*	<12 UI/l
*β*2GP-I antibodies (IgG)	42–44*	<10 UI /l

FT4: free thyroxin; TSH: Thyroid-stimulating hormone; *β*2GP-I: anti-*β*2-Glycoprotien-I.

*At control, three months after.

**Table 2:** Clinical and laboratory criteria of antiphospholipid antibody syndrome [6].

Clinical criteria	(1) Vascular thrombosis: one or more clinical episodes of arterial, venous, or small vessel thrombosis, in any tissue or organ
	(2) Pregnancy morbidity: one or more unexplained deaths of beyond the 10th week of gestation, one or more premature births of a morphologically normal neonate before the 34th week of gestation, or three or more unexplained consecutive spontaneous abortions before the 10th week of gestation

Laboratory criteria	(1) Lupus anticoagulant present in plasma, on two or more occasions at least 12 weeks apart
	(2) Anticardiolipin antibody of IgG and/or in high titer >40 GPL or MPL
	(3) Anti-*β*2-glycoprotein-I antibody of IgG and/or IgM isotype present on two or more occasions
